# Potential consequences of expanded MUAC-only programs on targeting of acutely malnourished children and ready-to-use-therapeutic-food allocation: lessons from cross-sectional surveys

**DOI:** 10.1186/s40795-019-0328-1

**Published:** 2020-02-10

**Authors:** Benjamin Guesdon, Alexia Couture, Danka Pantchova, Oleg Bilukha

**Affiliations:** 1 Action Contre La Faim, France, 14-16 Boulevard Douaumont, 75854 Paris, France; 2 Emergency Response and Recovery Branch, Division of Global Health Protection, Center for Global Health, Centers for Disease Control, 1600 Clifton Road, Atlanta, GA 30329 USA

**Keywords:** Wasting, Survey, Nutrition, Humanitarian, RUTF, MUAC, WHZ

## Abstract

**Background:**

Some of the recently piloted innovative approaches for the management of acute malnutrition in children use the “expanded MUAC-only” approach, with Mid Upper Arm Circumference (MUAC) < 125 mm as the sole anthropometric criterion for screening and admission, classification of cases as severe using the 115 mm cut-off, and use Ready-to-Use-Therapeutic-Food (RUTF) for the management of both moderate (MAM) and severe (SAM) cases of acute malnutrition. Our study aimed at exploring the potential consequences of this “expanded MUAC-only” program scenario on the eligibility for treatment and RUTF allocation, as compared with the existing WHO normative guidance.

**Methods:**

We analyzed data from 550 population representative cross-sectional cluster surveys conducted since 2007. We retrieved all children classified as SAM and MAM according to currently used case definitions, and calculated the proportions of SAM children who would be excluded from treatment, misclassified as MAM, or whose specific risks (because of having both MUAC and weight-for height deficits) would be ignored. We also analyzed the expected changes in the number and demographics (sex, age) of children meant to receive RUTF according to the new approach.

**Results:**

We found that approximately one quarter of SAM children would not be detected and eligible for treatment under the “expanded MUAC-only” scenario, and another 20% would be classified as MAM. A further 17% of the total SAM children would be admitted and followed only according to their MUAC or oedema status, while they also present with a severe weight-for height deficit on admission. Considering MAM targeting, about half of the MAM children would be left undetected. This scenario also shows a 2.5 time increase in the number of children targeted with RUTF, with approximately 70% of MAM and 30% of SAM cases among this new RUTF target.

**Conclusions:**

This empirical evidence suggests that adoption of “expanded MUAC-only” programs would likely lead to a priori exclusion from treatment or misclassifying as MAM a large proportion of SAM cases, while redirecting programmatic costs in favor of those less in need. It underscores the need to explore other options for improving the impact of programs addressing the needs of acutely malnourished children.

## Background

According to most recent global estimates, 7.3% or 49 million children under 5 were affected by wasting in 2018. Among them, nearly 17 million were severely wasted [1]. These children are at a higher risk of death than their well-nourished and healthy peers [2, 3]. About half to one million deaths each year have been attributed to wasting in children under 5 in past publications [4, 5].

WHO guidance for children suffering from Severe Acute Malnutrition (SAM), which includes both severe wasting and nutritional oedema (kwashiorkor), acknowledges that these children have an elevated risk of death and require intensive nutritional and medical support [6]. They should be screened, referred, and enrolled into an appropriate therapeutic feeding program. Current recommendations for these programs are to follow an inpatient treatment protocol when the patients present concomitant medical complications and an outpatient protocol when the patients have good appetite and no signs of complications [4, 6–8]. In practice, most children with SAM in the community are free from major medical complications and thus can be treated as outpatients [9].

Outpatient SAM management guidance for children aged 6–59 months comprises, alongside a systematic medical treatment and a weekly clinical and growth check-up, the provision of a specifically designed lipid-based nutritional product called Ready-to-Use-Therapeutic-Food (RUTF) [4]. RUTF is meant to optimize nutritional recovery by replacing the entire diet of the child, except for breast milk, for several weeks or months, until anthropometric deficits are corrected and oedema disappears. Although the entire package described above is required for an effective outpatient SAM management [10], RUTF-related costs for procurement, transport, storage, and distribution represent a large proportion of the costs of SAM management programs [11, 12]. A UNICEF 2013 evaluation of Community-based Management of Acute Malnutrition (CMAM) found that the cost of RUTF accounted for 50% of CMAM operating costs in five case study countries [13].

For moderate wasting, also called Moderate Acute Malnutrition (MAM), WHO also recommends screening, referral and adequate outpatient management including medical interventions (when necessary) and nutritional counselling. WHO, however, does not recommend that supplementary foods be provided as a default component of treatment for MAM [14]. Other UN agencies further endorsed this recommendation: not every child with MAM in every context requires this specific intervention, and there is also a concern about the association between rapid weight gain in childhood, including in the first 3 years of life, and the rising prevalence of overweight, obesity and non-communicable diseases (NCD) in later life, even in settings where undernutrition is prevalent [15]. It is acknowledged however that there might be a role for the provision of supplementary foods in settings where there is a high prevalence of wasting or food insecurity. Use and composition of supplementary foods for the management of moderate acute malnutrition currently follows this existing guidance [16, 17].

Internationally agreed upon case definitions for SAM include both low Weight-for-Height (WHZ < -3) and low Mid-Upper-Arm-Circumference (MUAC< 115 mm), as well as nutritional oedema [6]. For MAM, both − 3 ≤ WHZ < -2 and 115 mm ≤ MUAC< 125 mm are accepted as independent criteria [16, 18]. WHZ and MUAC indicators identify different populations of children as wasted. A recent analysis of more than 1800 cross-sectional surveys from 47 countries showed that only 16.5% of SAM children fulfilled both diagnostic criteria (MUAC < 115 mm and WHZ < -3) [19]. Re-analysis of past or modern datasets have also demonstrated that SAM children with low WHZ and SAM children with low MUAC have a similar risk of dying [3, 20, 21]. These studies also revealed that both deficits have additive effects on mortality risk: SAM children combining a low MUAC and a low WHZ, as well as those combining oedema and low WHZ, have a significantly higher risk of death. Although MUAC measurements are logistically easier, especially in community screenings, the need to use both WHZ and MUAC criteria independently for identification of SAM and MAM and admission to treatment is explicitly mentioned in the international normative guidance [8].

However, the use of MUAC as the only criterion for case finding and admission to therapeutic feeding programs has been increasingly promoted and applied in recent years [22–24]. A new set of suggestions for simplifications of the international guidance on SAM and MAM management is now proposing to implement “expanded MUAC-only” programs. In such programs, screening, admission and discharge criteria would be based solely on MUAC or oedema (e.g. WHZ status of the child would not be assessed nor considered); all children with MUAC < 125 mm would be enrolled in the program; children with MUAC < 115 or nutritional oedema would be classified as SAM, those with 115 ≤ MUAC< 125 would be classified as MAM, and they would be treated with different doses of RUTF [17, 25].

Although the “expanded MUAC-only” scenario greatly simplifies guidance for SAM and MAM treatment, it raises a range of concerns. Some of these concerns relate to targeting. Since screening and admission criteria proposed in “expanded MUAC-only” programs are based solely on MUAC or oedema, these programs indeed entail (1) exclusion of SAM children (WHZ < -3) with MUAC≥125 mm, (2) exclusion of MAM children (− 3 ≤ WHZ < -2) with MUAC≥125 mm, (3) treatment of SAM children with WHZ < -3 and 115 ≤ MUAC< 125 mm as MAM children, (4) inability to identify and adequately follow SAM children with both WHZ and MUAC deficits (or children with WHZ < -3 and oedema). There is also a question whether the high cost of RUTF provision to MAM children is justified by need since a large number of MAM children, many of whom may not require such a specific and costly support as per current international recommendations, would receive RUTF. That would take place while a substantial proportion of SAM children, for which RUTF was initially designed, might be excluded or undertreated.

After consideration of recently piloted program simplifications approaches, including the “expanded MUAC-only” program scenario at the recent WHO/UNICEF/UNHCR/WFP expert consultation in March of 2019, the conclusion was that more evidence is needed on potential implications of this approach for the coverage, effectiveness, cost and impact of treatment of child wasting, including in exceptional circumstances [26]. Therefore, this study was undertaken to explore the potential consequences of “expanded MUAC-only” program scenario on the number and percentage of SAM and MAM children either excluded from treatment or misclassified and the implications on the number, demographic and nutritional profile of the children targeted by RUTF provision, as compared with current normative guidance.

## Methods

Data for these analyses were obtained from Action Contre la Faim (ACF) International, an international humanitarian non-governmental organization that conducts multiple field nutrition surveys in humanitarian settings worldwide [27]. Surveys conducted during 2007–2018 that measured both sex, age, height, weight, oedema and mid-upper arm circumference (MUAC) in children aged 6–59 months were included. All surveys included were population representative cross-sectional two-stage cluster surveys following standard survey and sampling procedure and usually conducted at the district level [28].

Survey countries were grouped into six geographic categories: Latin America and the Caribbean; East and South Africa; Democratic Republic of Congo (DRC); West and Central Africa; East Asia and Pacific; and South Asia [29]. DRC was kept as its own category due to the large number of surveys from the country. Countries that had fewer than five surveys conducted during 2007–2018 were excluded from the analyses. We considered that fewer surveys would have too few cases of acute malnutrition to produce reliable per-country estimates. The Middle East and North Africa regions were not included since none of the countries had five or more surveys conducted during the study period.

Weight-for-height Z scores (WHZ) were calculated for all children using the WHO SAS macro, which applies the WHO 2006 growth standards [30]. Children with missing data for age, sex, weight, height or MUAC and with age out of range (6.0–59.99 months) were excluded. Children were also excluded following WHO flagging criteria if they had WHZ that fell outside of +/− 5 Z-scores.

Acute malnutrition was defined as either by MUAC only, by WHZ only, or by both criteria (MUAC and/or WHZ). Severe malnutrition (SAM) defined by MUAC (SAMmuac) was MUAC < 115 mm and/or clinical signs of oedema, and moderate acute malnutrition (MAM) defined by MUAC (MAMmuac) was 115 mm ≤ MUAC < 125 mm. SAM defined by WHZ (SAMwhz) was WHZ < − 3, and MAM by WHZ (MAMwhz) was − 3 ≤ WHZ < − 2. SAM defined by MUAC and/or WHZ (SAMall) was MUAC < 115 m and/or WHZ < − 3 and/or clinical signs of oedema, and MAM defined by MUAC and/or WHZ (MAMall) was 115 mm ≤ MUAC < 125 mm and and/or − 3 ≤ WHZ < − 2. Global acute malnutrition (GAM) included both MAM and SAM.

**Fig. 1:**
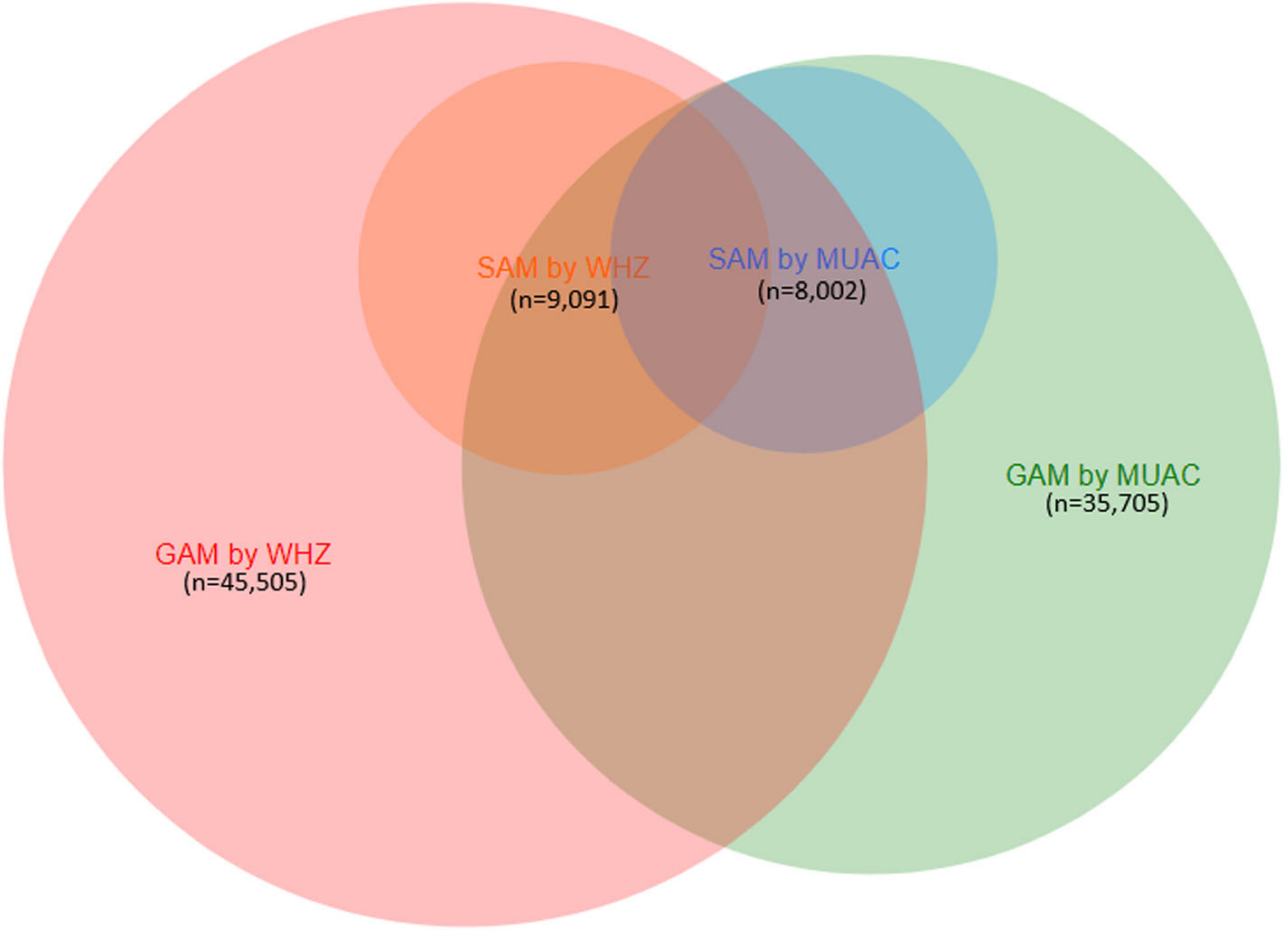
Overlap of the SAM and GAM categories of children defined either by WHZ or MUAC criteria based on the aggregated totals presented in Table 1 *SAM* severe acute malnutrition, *GAM* global acute malnutrition, *RUTF* ready-to-use therapeutic food, *WHZ* weight-for-height z-score, *MUAC* mid-upper arm circumference Categories represented by colors are as follows: GAM by WHZ (*red*), SAM by WHZ (*orange*), SAM by MUAC (*blue*), and GAM by MUAC (*green*)

To explore the effects of the “expanded MUAC-only” programming on the targeting of SAM children, we considered the following categories of children (see Fig. 1 for visualization of categories defined below):

1. “Excluded SAM” – proportion of SAMall children not detected and not eligible for treatment (WHZ < -3 and MUAC > 125 mm and no oedema).
2. “Underestimated SAM” – proportion of SAMall children detected but classified as MAM (WHZ < -3 and 115 ≤ MUAC< 125 mm and no oedema).
3. “Ignored Risk SAM” – proportion of SAMall children detected, without adequate consideration for their increased risks and needs, and without subsequent WHZ follow-up (WHZ < -3 and MUAC< 115 mm; or WHZ < -3 and oedema).
4. “Correctly Detected SAM” – proportion of SAMall children classified as SAM in accordance with standard WHO recommendations (MUAC < 115 mm and/or oedema and WHZ ≥ -3).

We also generated the categories to explore effects of the “expanded MUAC-only” programming on the targeting of MAM children and program implications. They were as follows:

1. “Excluded MAM” – proportion of MAMall children not detected and admitted for treatment (WHZ < -2 and > − 3 and MUAC > 125 mm and no oedema).
2. “Proportion of MAM in the program” – proportion of MAMall children among all children in new program.
3. “RUTF allocation increase” – ratio of expected increase of children receiving RUTF, assuming the “new” target for RUTF-based treatment includes all children identified as SAMmuac and MAMmuac, and the “old” target includes SAMall children.

For country- and region-specific analyses, we aggregated all child counts from individual surveys by country and then calculated proportions and ratios.

Further, to describe basic demographics of children in each of the four SAM (i.e., “excluded,” “underestimated,” “ignored risk,” and “correctly detected”) and two MAM (“excluded,” “included”) groups defined above, we calculated (1) the proportion of females and (2) the proportion of younger children (aged 6–23 months) in each of these groups.

This study was determined as non-research by the institutional review board of the Centers for Disease Control and Prevention since it entailed secondary analysis of routinely collected programmatic data. No individual identifiers were included in the dataset used for analysis. Data were aggregated, cleaned and analyzed using SAS Version 9.4 and RStudio [31, 32].

## Results

Final analyses included 550 surveys implemented in 22 countries, which included over 400,000 children aged 6–59 months (Table 1). A schematic representation reflecting different groups of SAM and MAM children identified by WHZ and MUAC, as well as their overlap, is shown as a venn diagram (Fig. 1). The areas of various groups represented in Fig. 1 are proportional to their size as overall aggregate total in Table 1. Percentage of SAM or MAM children who would be excluded or underestimated, MAM vs. SAM status of children who would receive RUTF, and the times increase in the number of children who would be treated with RUTF under an “expanded MUAC-only” scenario, as compared with current normative guidance, are described by region and country in Table 1.

**Table 1:** Description of surveys included in the sample and distribution of SAM and MAM children 6 to 59 months among the categories defined based on “expanded MUAC-only” admission criteria, by country and region

Region	Country	N surveys	N children	SAMall (%)	MAMall (%)	Excluded SAM (%)	Under-estimated SAM (%)	Ignored Risk SAM (%)	Correctly Detected SAM (%)	Excluded MAM (%)	Prop. MAM in Program (%)	RUTF Allocation Increase
East Asia and Pacific	Myanmar	10	5469	4.0%	17.5%	20.6%	26.6%	21.1%	31.7%	52.8%	72.3%	2.86
	Philippines	5	3202	1.4%	5.8%	59.1%	20.5%	2.3%	18.2%	85.9%	59.1%	1.00
	**Total**	**15**	**8671**	**3.0%**	**13.1%**	**27.1%**	**25.6%**	**17.9%**	**29.4%**	**58.2%**	**71.4%**	**2.55**
Latin America and Caribbean	**Haiti**	**24**	**13,156**	**1.2%**	**4.8%**	**28.4%**	**14.2%**	**21.6%**	**35.8%**	**49.8%**	**73.1%**	**2.67**
South Asia	Afghanistan	57	42,241	4.2%	11.2%	25.5%	12.7%	12.8%	49.0%	32.1%	70.7%	2.54
	Bangladesh	37	18,181	2.4%	13.4%	34.1%	29.3%	19.2%	17.4%	67.3%	73.4%	2.48
	India	9	3861	5.6%	24.2%	36.7%	37.6%	14.7%	11.0%	70.7%	66.4%	1.89
	Nepal	10	5511	4.6%	16.7%	19.1%	31.3%	23.4%	26.2%	52.8%	67.7%	2.50
	Pakistan	19	12,850	6.4%	17.0%	25.3%	19.0%	23.1%	32.6%	49.9%	64.2%	2.09
	**Total**	**132**	**82,644**	**4.2%**	**13.5%**	**26.8%**	**19.1%**	**16.9%**	**37.2%**	**48.2%**	**69.3%**	**2.39**
East and South Africa	Kenya	36	22,622	2.4%	13.7%	54.4%	20.3%	9.3%	16.1%	78.7%	72.6%	1.66
	Madagascar	9	4330	2.8%	9.2%	11.7%	9.2%	22.5%	56.7%	34.8%	71.0%	3.04
	Somalia	7	5087	4.5%	18.5%	34.2%	24.6%	10.5%	30.7%	49.9%	75.9%	2.73
	South Sudan	26	15,166	5.2%	19.2%	39.6%	27.9%	14.3%	18.2%	67.4%	66.5%	1.80
	Sudan	41	35,100	4.7%	17.4%	34.0%	26.3%	19.4%	20.4%	65.0%	66.2%	1.95
	Uganda	19	21,619	2.6%	9.9%	18.8%	21.4%	17.0%	42.8%	36.6%	74.5%	3.19
	**Total**	**138**	**103,924**	**3.8%**	**15.0%**	**35.1%**	**24.4%**	**16.2%**	**24.3%**	**62.6%**	**69.7%**	**2.14**
West and Central Africa	Burkina Faso	7	5355	4.1%	12.9%	14.0%	28.4%	33.3%	24.3%	55.0%	61.9%	2.26
	Central African Republic	13	8607	2.5%	8.5%	11.1%	14.7%	27.2%	47.0%	29.5%	72.9%	3.28
	Chad	18	10,992	5.7%	16.5%	22.0%	27.1%	24.0%	26.9%	49.5%	65.3%	2.25
	Guinea	5	4006	3.1%	7.8%	11.9%	23.0%	31.7%	33.3%	41.9%	62.1%	2.33
	Mali	13	9108	2.7%	11.4%	34.1%	25.3%	21.7%	18.9%	64.5%	69.3%	2.14
	Niger	10	6976	3.6%	15.0%	16.3%	26.3%	27.5%	29.9%	43.5%	73.8%	3.19
	Nigeria	5	2642	6.9%	15.0%	27.1%	15.5%	22.7%	34.8%	52.9%	58.5%	1.76
	Sierra Leone	16	9759	1.5%	5.1%	34.5%	16.2%	9.9%	39.4%	59.4%	68.5%	2.08
	**Total**	**87**	**57,445**	**3.5%**	**11.4%**	**21.4%**	**23.5%**	**24.9%**	**30.2%**	**49.9%**	**67.4%**	**2.41**
DRC		**154**	**134,298**	**3.6%**	**10.6%**	**19.2%**	**14.1%**	**13.4%**	**53.3%**	**35.6%**	**70.2%**	**2.72**
Overall Aggregate Total		**550**	**400,138**	**3.7%**	**12.3%**	**25.8%**	**19.6%**	**16.7%**	**37.9%**	**49.6%**	**69.6%**	**2.44**

*SAM* severe acute malnutrition, *MAM* moderate acute malnutrition, *RUTF* ready-to-use therapeutic food, *DRC* Democratic Republic of Congo

Regional and overall totals are in bold

Considering targeting of SAM children, overall aggregated counts demonstrate that under “expanded MUAC-only” scenario, around 26% of SAM children would not be detected and eligible for treatment, and another 20% would be classified as MAM. A further 17% of the total SAM caseload would be admitted and followed only according to their MUAC or oedema status, while they also present with a WHZ < -3 on admission. Only 38% of SAM caseload would be correctly identified and treated as MUAC-only SAM cases (MUAC< 115 mm or oedema and WHZ ≥ -3) (Table 1). Considering targeting of MAM children, approximately half of the MAM caseload would be missed under “expanded MUAC-only” scenario (Table 1). Considering SAM vs. MAM status of the children receiving RUTF under this scenario, about 70% would be MAM cases. Because of the extension of the RUTF provision to MAM children, there would be an approximately 2.5 times increase in the number of children receiving RUTF under the “expanded MUAC-only” scenario as compared to current normative guidance.

**Fig. 2:**
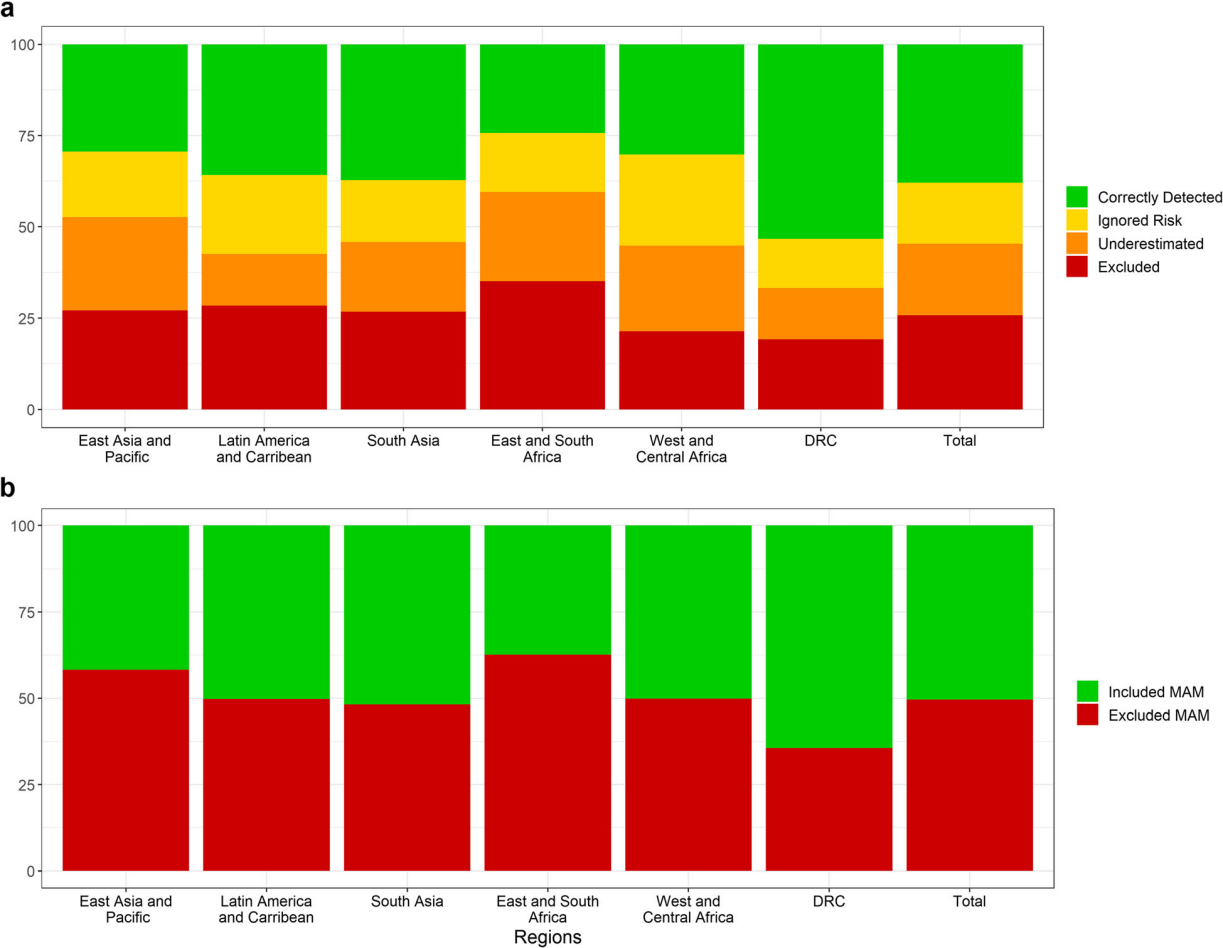
Distribution of SAM (**a**) and MAM (**b**) children among the categories defined based on “expanded MUAC-only” admission criteria, by region *SAM* severe acute malnutrition, *MAM* moderate acute malnutrition, *RUTF* ready-to-use therapeutic food, *DRC* Democratic Republic of Congo Categories represented by colors in **a** are as follows: Correctly Detected (*green*), Ignored Risk (*yellow*), Underestimated (*orange*), and Excluded (*red*). Categories represented by colors in **b** are as follows: Included MAM (green) and Excluded MAM (*red*)

**Fig. 3:**
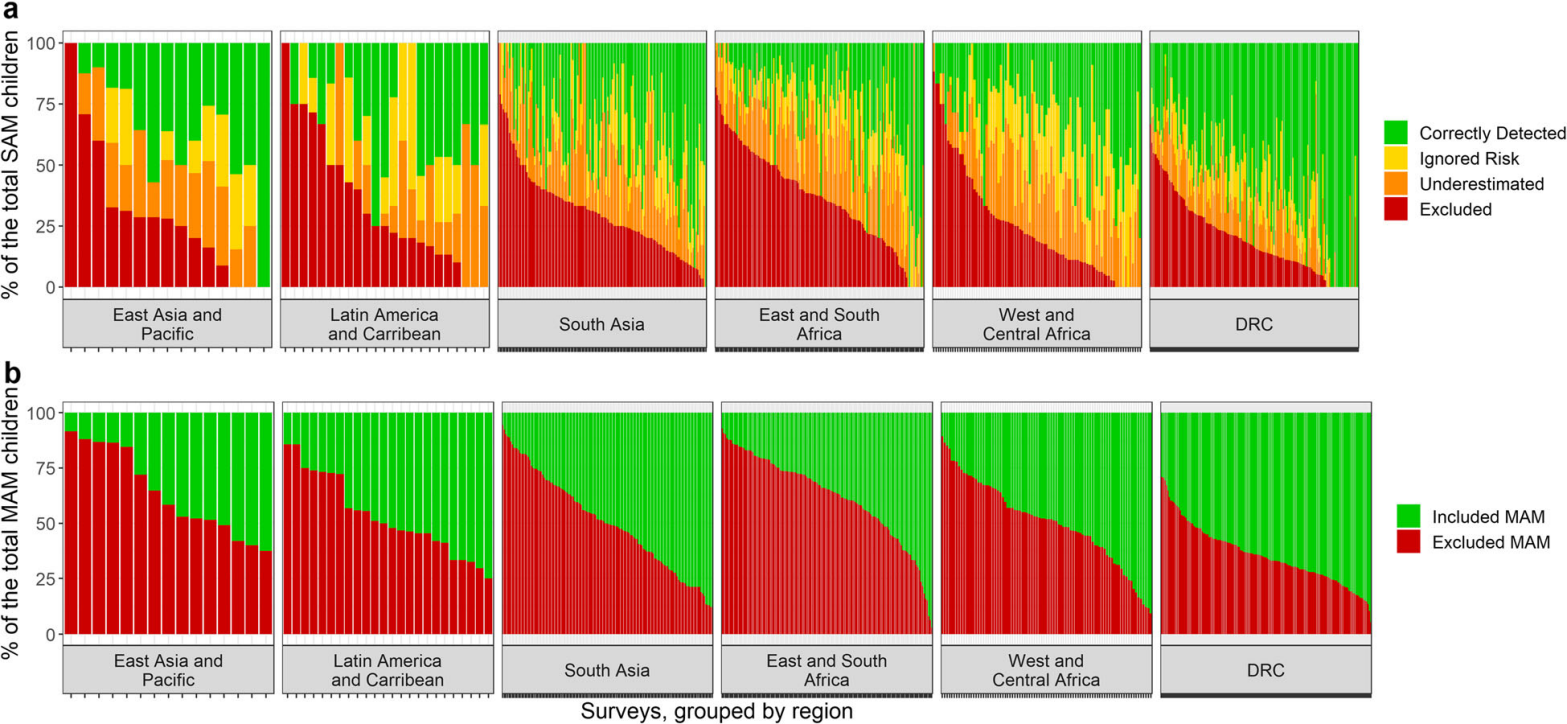
Distribution of SAM (**a**) and MAM (**b**) children among the categories defined based on “expanded MUAC-only” admission criteria in individual surveys, grouped by region *SAM* severe acute malnutrition, *MAM* moderate acute malnutrition, *RUTF* ready-to-use therapeutic food, *DRC* Democratic Republic of Congo Categories represented by colors in **a** are as follows: Correctly Detected (*green*), Ignored Risk (*yellow*), Underestimated (*orange*), and Excluded (*red*). Categories represented by colors in **b** are as follows: Included MAM (*green*) and Excluded MAM (*red*)

These figures are broadly consistent across regions, although it can be noted that East and South Africa as well as East Asia and Pacific regions show the highest proportion of excluded SAM and MAM children, whereas DRC shows the lowest proportion of these categories (Fig. 2). Country-specific data show the proportion of excluded SAM and MAM cases as well as undertreated SAM cases is the highest in the countries with the highest caseload (i.e. India, Bangladesh), or in some of those affected by chronic or acute crises, such as South Sudan and Somalia. As shown in Fig. 3, a considerable variability in these proportions exists among survey populations even from the same region or country.

Proportions of children under 2 years of age and females within each category of SAM and MAM described above are shown by region and country in Table 2. A consistent pattern observed across all regions is that SAM and MAM groups who would be excluded from treatment under an “expanded MUAC-only” program scenario have a lower percentage of young children and lower proportion of females than those included. While young children and females represent a minority of approximately 30 and 40% (respectively) of the populations of SAM and MAM children excluded, they represent approximately 70 and 60% (respectively) of the populations of SAM and MAM children correctly detected under this scenario.

**Table 2:** Proportion of children under 2 years of age and females in SAM and MAM categories defined based on “expanded MUAC-only” admission criteria, by country and region

Region	Country	Excluded SAM (< 2 years) (%)	Under- estimated SAM (< 2 years) (%)	Ignored Risk SAM (< 2 years) (%)	Correctly Detected SAM (< 2 years) (%)	Excluded MAM (< 2 years) (%)	Included MAM (< 2 years) (%)	Excluded SAM (females) (%)	Under- estimated SAM (females) (%)	Ignored Risk SAM (females) (%)	Correctly Detected SAM (females) (%)	Excluded MAM (females) (%)	Included MAM (females) (%)
**East Asia and Pacific**	Myanmar	11.1%	62.1%	73.9%	82.6%	29.0%	74.3%	26.7%	37.9%	52.2%	65.2%	41.3%	64.1%
	Philippines	34.6%	77.8%	100.0%	25.0%	37.7%	92.3%	42.3%	55.6%	0.0%	75.0%	45.3%	69.2%
	**Total**	**19.7%**	**64.2%**	**74.5%**	**76.6%**	**31.1%**	**75.3%**	**32.4%**	**40.3%**	**51.1%**	**66.2%**	**42.2%**	**64.4%**
**Latin America and Caribbean**	**Haiti**	**39.1%**	**73.9%**	**77.1%**	**70.7%**	**40.1%**	**68.7%**	**50.0%**	**17.4%**	**57.1%**	**58.6%**	**35.7%**	**58.2%**
**South Asia**	Afghanistan	44.3%	69.0%	88.1%	82.7%	43.9%	74.3%	39.9%	43.8%	44.9%	64.3%	39.3%	57.5%
	Bangladesh	25.5%	61.7%	89.3%	89.5%	29.1%	80.0%	38.9%	43.0%	50.0%	81.6%	38.7%	65.2%
	India	22.5%	56.1%	78.1%	83.3%	27.6%	71.1%	36.3%	39.0%	56.3%	66.7%	42.0%	64.1%
	Nepal	30.6%	61.3%	85.0%	92.5%	34.0%	74.2%	34.7%	35.0%	50.0%	70.1%	40.5%	61.8%
	Pakistan	28.5%	66.5%	89.4%	78.2%	28.5%	65.2%	39.6%	32.3%	46.6%	66.9%	39.6%	56.7%
	**Total**	**35.3%**	**64.5%**	**87.8%**	**82.7%**	**33.4%**	**73.2%**	**39.1%**	**39.3%**	**47.3%**	**66.2%**	**39.6%**	**59.0%**
**East and South Africa**	Kenya	11.1%	46.8%	80.4%	79.5%	18.4%	70.7%	41.6%	37.8%	31.4%	67.0%	41.5%	63.3%
	Madagascar	14.3%	54.5%	74.1%	82.4%	23.2%	64.9%	28.6%	27.3%	33.3%	55.9%	34.8%	52.9%
	Somalia	29.5%	69.6%	87.5%	90.0%	23.8%	81.1%	42.3%	35.7%	37.5%	62.9%	40.2%	55.9%
	South Sudan	24.5%	61.1%	77.9%	78.5%	25.6%	69.7%	43.3%	38.9%	51.3%	62.5%	44.4%	57.4%
	Sudan	20.5%	66.6%	83.4%	84.9%	24.8%	75.3%	33.7%	31.3%	47.5%	65.6%	41.2%	59.9%
	Uganda	42.1%	68.9%	84.5%	82.0%	46.4%	75.7%	43.9%	34.4%	45.4%	67.2%	39.6%	56.7%
	**Total**	**21.5%**	**63.4%**	**82.1%**	**82.9%**	**25.0%**	**74.0%**	**38.8%**	**34.5%**	**45.6%**	**64.8%**	**41.7%**	**58.5%**
**West and Central Africa**	Burkina Faso	54.8%	77.8%	74.3%	79.6%	52.5%	68.1%	19.4%	14.3%	47.3%	68.5%	39.1%	63.2%
	Central African Republic	25.0%	56.3%	71.2%	63.7%	29.0%	60.6%	45.8%	34.4%	44.1%	58.8%	39.6%	54.2%
	Chad	30.7%	58.6%	74.0%	78.6%	22.7%	67.6%	39.4%	40.8%	50.7%	69.6%	39.6%	60.3%
	Guinea	60.0%	65.5%	92.5%	85.7%	46.6%	78.6%	33.3%	41.4%	42.5%	61.9%	40.5%	60.4%
	Mali	44.7%	73.0%	83.3%	76.6%	36.2%	75.1%	22.4%	25.4%	53.7%	68.1%	43.9%	60.5%
	Niger	31.7%	69.7%	72.5%	92.0%	36.3%	73.6%	31.7%	25.8%	37.7%	78.7%	31.4%	63.2%
	Nigeria	40.8%	57.1%	80.5%	69.8%	38.8%	62.4%	40.8%	46.4%	48.8%	63.5%	39.7%	68.3%
	Sierra Leone	38.8%	91.3%	100.0%	80.4%	51.7%	82.7%	42.9%	26.1%	35.7%	46.4%	36.1%	60.9%
	**Total**	**38.1%**	**66.4%**	**77.2%**	**77.4%**	**35.9%**	**69.7%**	**34.6%**	**32.3%**	**46.7%**	**65.4%**	**39.0%**	**60.7%**
**DRC**		**27.5%**	**44.7%**	**65.9%**	**72.3%**	**27.1%**	**65.4%**	**37.9%**	**35.9%**	**42.3%**	**57.0%**	**39.9%**	**55.4%**
**Overall Aggregate Total**		**28.4%**	**59.8%**	**78.0%**	**77.1%**	**29.1%**	**70.0%**	**38.2%**	**35.6%**	**45.6%**	**61.5%**	**40.4%**	**57.9%**

*SAM* severe acute malnutrition, *MAM* moderate acute malnutrition, *RUTF* ready-to-use therapeutic food, *DRC* Democratic Republic of Congo

Regional and overall totals are in bold

## Discussion

As demonstrated in this study, analyses of relative proportions of SAM and MAM cases identified by MUAC vs. WHZ criteria in the community is critical in the investigation of the expected consequences of an “expanded MUAC-only” programming scenario on targeting. Through our secondary analysis of a large number of cross-sectional surveys, we identified two important potential drawbacks of the “expanded MUAC-only” scenario.

First, our analyses demonstrates substantial decrease in the number of targeted SAM children, as compared with the population of children who are severely acutely malnourished and eligible for rehabilitative nutrition programs. A switch to “expanded MUAC-only” programs would lead to situations where a large proportion of SAM children and families in need would be denied access to these services. Of critical concern is the fate of the 50% SAM children, at high risk of death, who would be deemed ineligible for treatment or receive sub-standard treatment under an “expanded MUAC-only” scenario. In addition, about 50% of MAM children in the community would also be excluded a priori from these programs. The first drawback to expect is thus a large a priori restriction of the coverage of SAM and MAM cases.

Second, our results show a seemingly counterintuitive (given reduction in target discussed above) increase of the number of children who would be eligible to receive RUTF as part of their treatment. This is the result of a change in the population eligible for RUTF-based treatment, from the current target population (all SAM children) to all children with MUAC< 125 mm. SAM children would constitute approximately one-third of this “new target” population and the rest (around 70%) would be comprised of MAM children. Even though a lower RUTF dosage is intended for MAM cases under this scenario, still a large part of the RUTF-related costs, from purchase to end-user distribution will be allocated to MAM children. Considering the current situation where RUTF-related costs, which constitute a large proportion of therapeutic feeding program costs, are one of the key barriers for the scaling-up of these therapeutic feeding programs and their sustainability [13, 33], the universal expansion of RUTF use for MAM children would be a programmatic challenge. Since this expansion would occur at the same time as RUTF is denied or restricted for many SAM children, the planned increase in the level of support for MAM children may ultimately take place at the expense of SAM children. The second drawback to expect is thus a potentially inequitable use of the costly RUTF resource, outside its initial (and principal) indication.

It has been argued that SAM children presenting with a WHZ < -3 but a MUAC> = 115 mm are relatively healthy, and that SAM children with a low MUAC have a higher risk of death [22, 34]. These hypotheses are being challenged by a range of clinical studies [35, 36], as well as by direct observation of mortality risks in cohorts of patients [20] and in large community cohorts [3]. It appears that the initially formulated hypothesis that SAM children with low WHZ are at lower mortality risk than low MUAC SAM children was driven by analyses affected by Simpson’s paradox, comparing populations of cases not appropriately disaggregated [20]. The recent evidence suggests that SAM children with low WHZ and those with low MUAC have similar risk of death [3, 20]. This evidence also shows that children presenting with both deficits (low MUAC and low WHZ) or with combined low WHZ and oedema display much higher risk of mortality than children with only one deficit (low WHZ or low MUAC). Arguing in favor of the prioritization of these small subpopulations of SAM cases with both deficits or with combined low WHZ and oedema seems justified, especially in situations of dramatically constrained resources, such as sudden RUTF shortages or at the onset of an acute crisis. The identification and adequate treatment of this subpopulation of SAM cases in such exceptional resource-restricted situations would require the measurement of WHZ alongside MUAC and oedema. Although “expanded MUAC-only” programs would include this subpopulation of SAM children, these children would remain unidentified (since WHZ is not measured), and their specific elevated risks would go unaddressed. Further, under “expanded MUAC-only” programs, these children may be discharged early, without reaching the minimal WHZ level indicative of recovery [37].

Besides simple exclusion, the “expanded MUAC-only” program would also classify a large part of the population of SAM cases as MAM. The expected consequence of this would be to treat them with a lower RUTF dose, without routine medical treatment, and with a lesser level of medical assessment and follow-up, which may affect treatment outcomes Although currently available evidence on this topic is limited, a RUTF dose meeting total daily nutritional requirements may improve recovery and prevent relapse compared to RUTF given as a supplement to the usual diet [38]. Recent studies involving uncomplicated SAM patients reported important incidence of co-morbidities and referrals to hospital during the treatment period, thereby highlighting the importance of keeping these patients under close medical attention [39, 40]. Failure to adequately address co-morbidities occurring during outpatient treatment has been highlighted as important risk factors of poor treatment outcomes [41]. Accordingly, randomized controlled trials comparing treatment outcomes with or without systematic antibiotic treatment showed that this component of SAM outpatient therapeutic feeding protocols is also required to prevent medical complications and deaths during treatment [8, 36, 42, 43]. As summarized by Bhutta and colleagues, all components of care are required to ensure optimal treatment outcomes, beyond the choice of food commodity [10]. Therefore, adverse consequences on treatment outcomes can be expected when SAM cases are misclassified, underestimated, and subsequently undertreated as MAM.

It is difficult to provide relevant estimations of the negative consequences of excluding 50% of the MAM caseload, i.e. those children with WHZ < -2 and MUAC≥125 mm. Both the exact levels of morbidity and mortality risk, the physiological needs of MAM children, as well as the best way to promote their recovery, are not well established [15, 44]. However, these MAM children who would be excluded from “expanded MUAC-only” programs represent the bulk of the United Nation agencies’ joint estimates of global wasting caseload, since only WHZ < -2 indicator is used to produce these estimates [45].

The basic demographic profile of the SAM and MAM children show that while the majority of those included in the MUAC-only program would be young (< 2 years) children and females, about 30% of the children excluded from treatment would be younger than 2 years and about 40% would be female. This is in line with what we know about the usual distribution of age and sex in the population of children with a MUAC below an absolute cut-off, as compared with the population of children with a low WHZ and a MUAC above the cut-off [6, 46]. The clinical significance of this difference in age and sex between excluded and included SAM and MAM children is not straightforward. On the one hand, it has been argued that younger children and girls have a higher risk of death among malnourished children and that the proportion of cases with these characteristics would further indicate that low MUAC children are more at risk than the other SAM children [22]. On the other hand, it has long been demonstrated that young children and girls have lower MUAC values than older children and boys because they are smaller, and this alone does not necessarily signify the higher mortality risk. This rather means that a lower level of anthropometric deficits is required for them to fall under an absolute MUAC cut-off than for older children and boys [47]. While this explains the higher proportion of young children and girls among children classified as SAM or MAM according to an absolute MUAC value, it may also indicate lower levels of nutritional impairments and lower associated risk of death in these subcategories of low MUAC SAM children. A recent analysis of mortality risks associated with anthropometric deficits further confirmed this hypothesis by showing a lower increase in the risk of death associated with MUAC< 115 mm in young children than in older children [3]. In that study, sex did not appear to have a role in the mortality risk associated with anthropometric deficits.

Of note, the difference in prevalence of wasting as assessed by WHZ versus the prevalence assessed by MUAC is greatest in crises when wasting by WHZ becomes more prevalent [48]. Drawbacks of the MUAC-only approach resulting in an underestimation of caseload and exclusion of those in need of treatment are thus expected to worsen in crisis, as confirmed by the field experiences in crises and higher caseload contexts [49]. Therefore, the higher the prevalence of wasting in the population (indicating a crisis), the higher proportion of MAM and SAM children will remain undetected by a MUAC-only program.

The rationale for the expansion of the use of RUTF to MAM children also warrants further discussion. Although positive impacts of lipid-based nutritional supplements on MAM recovery and weight gain have been reported in the past [44], considerable knowledge gaps remain. Of note, WHO recently recommended not to provide any nutritional products as a supplement for MAM children outside of exceptional circumstances [14]. First, it is unclear what the optimal requirements for MAM children are, and second, it is very likely that there would be options other than RUTF to adequately improve their diet [15, 50]. It has been proposed for instance that programmatic solutions relying on the improvement in complementary foods and child health through a more holistic approach would achieve similar results in the short term and better ones in the mid to long term. Concerns have also been raised about potentially deleterious impact of product-based approaches relying on high-fat and high-sugar processed foods at a time when the double burden of under- and overnutrition is increasingly threatening the health of low and middle income country populations [51].

The increase in the targeted number of children cannot be easily translated in terms of increase in quantity of RUTF, given the number of parameters which have to be taken into account but either remain unresolved (appropriate RUTF dosage for MAM, duration of treatment) or may be highly context-dependent (changes in coverage, logistical costs associated with RUTF supply and distribution). However, a large increase in RUTF-related costs (not only for purchase but also for transport and distribution) is likely to accompany the two- to three-fold increase in numbers of children eligible for RUTF support. Concerns have been raised about the use of RUTF outside its initial target, the conflicts of interests which may arise in this growing market, and opportunity cost of not directing resources for potentially better long-term investments [52–54]. Expanding the use of RUTF for the purpose of program simplification may provide additional arguments to this criticism.

There is no doubt that adoption of an “expanded MUAC-only” program scenario would lead to dramatic simplification of the current normative guidance for management of acute malnutrition and would address many of the barriers faced by program implementers and Ministries of Health in resource constrained environments. However, the potential limitations of targeting demonstrated in this study as well as potential consequences for coverage, effectiveness and cost-effectiveness of such programs may preclude others for considering such simplified approach outside of exceptional circumstances, where WHZ measurement for screening, admission and discharge purposes is simply not feasible. In such circumstances, “expanded MUAC-only” programs could be considered as a temporary option. Effectiveness and cost-effectiveness of this approach in exceptional circumstances should be further investigated and might ultimately be considered as inadequate.

A major strength of this study is the high number and quality of the cross-sectional surveys it builds upon. The 550 surveys contributing to the analysis are almost all surveys conducted by Action Against Hunger during the recent period 2007–2018. During these surveys, planning, data collection and analysis followed standardized methods embedding rigorous quality controls [28] and were supervised and validated a posteriori by highly qualified and trained staff. These surveys focused on countries where acute malnutrition is likely a problem and where feeding programs are implemented, including emergency contexts. This study however has several limitations. First, only 22 countries (those for which we had 5 or more surveys) were included. The disaggregation of the results by country and region is thus for illustration; these data cannot be considered as representative globally or regionally. Furthermore, surveys were mostly of small scale, with the objective of providing an accurate estimate of wasting at the district level. Thus, the results we obtained may not be representative of the country overall. We also demonstrate that large variability exists within countries and regions, so one cannot readily predict at the lower administrative level where we should expect more excluded children. Considering potential biases, it is important to mention that countries with more surveys have more influence on the regional or global estimates – for example, about 30% of included surveys are from DRC. In DRC, low MUAC or oedema detect higher proportions of SAM and MAM children compared with other regions. Considering that countries where these proportions are on the contrary very low, such as India or Bangladesh, contribute only 1.6 and 6.7% of the surveys we analyzed, while they are major contributors to the global caseload for acute malnutrition, the global consequences of the “expanded MUAC-only” program scenario on the exclusion or underestimation of acute malnutrition cases is likely underestimated.

## Conclusion

Although the need for simplification of nutrition treatment programs is well appreciated, our analyses from field surveys in countries where treatment programs have been implemented suggests that the adoption of “expanded MUAC-only” programs would likely lead to both excluding a large proportion of those most in need from receiving treatment, decreasing their level of support, and increasing by a large extent the use of RUTF for a population of children with a lesser level of vulnerability. Our study challenges the rationale behind MUAC-only programs and underscores the need to explore other solutions for improving the impact of programs aimed at addressing the needs of acutely malnourished children, such as innovations aimed at simplifying the detection of all SAM and MAM children in the community (including those with low WHZ), and innovations aimed at decreasing dependency on costly imported nutritional products.

## Data Availability

The data that support the findings of this study are available from Action Contre la Faim but restrictions apply to the availability of these data, which were used under license for the current study, and so are not publicly available. Data are however available from the authors upon reasonable request and with permission of Action Contre la Faim.

## Abbreviations

ACF: Action Contre le Faim
CMAM: Community-based Management of Acute Malnutrition
DRC: Democratic Republic of Congo
GAM: Global Acute Malnutrition
MAM: Moderate Acute Malnutrition
MUAC: Mid-upper arm circumference
NCD: Non-communicable diseases
RUTF: Ready-to-Use-Therapeutic-Food
SAM: Severe Acute Malnutrition
UNHCR: United Nations High Commissioner for Refugees
WFP: World Food Programme
WHO: World Health Organization
WHZ: Weight-for-height z-score
